# Seasonal reproduction leads to population collapse and an Allee effect in a stage-structured consumer-resource biomass model when mortality rate increases

**DOI:** 10.1371/journal.pone.0187338

**Published:** 2017-10-31

**Authors:** Zepeng Sun, André M. de Roos

**Affiliations:** Institute for Biodiversity and Ecosystem Dynamics, University of Amsterdam, Amsterdam, The Netherlands; Centre National de la Recherche Scientifique, FRANCE

## Abstract

Many populations collapse suddenly when reaching low densities even if they have abundant food conditions, a phenomenon known as an Allee effect. Such collapses can have disastrous consequences, for example, for loss of biodiversity. In this paper, we formulate a stage-structured consumer-resource biomass model in which adults only reproduce at the beginning of each growing season, and investigate the effect of an increasing stage-independent background mortality rate of the consumer. As the main difference with previously studied continuous-time models, seasonal reproduction can result in an Allee effect and consumer population collapses at high consumer mortality rate. However, unlike the mechanisms reported in the literature, in our model the Allee effect results from the time difference between the maturation of juveniles and the reproduction of adults. The timing of maturation plays a crucial role because it not only determines the body size of the individuals at maturation but also influences the duration of the period during which adults can invest in reproductive energy, which together determine the reproductive output at the end of the season. We suggest that there exists an optimal timing of maturation and that consumer persistence is promoted if individuals mature later in the season at a larger body size, rather than maturing early, despite high food availability supporting rapid growth.

## Introduction

Sudden collapses of populations increase the risk of population extinction and may hence result in the loss of biodiversity. In some cases population growth rate declines as density falls below a threshold [1], due to positive density dependence at low population density. Such a phenomenon, broadly referred to as an Allee effect, impedes population recovery from low densities. Allee effects are of great importance in fisheries because they increase the risk of collapses of fish populations owing to overfishing [2], and can slow down or even impede the recovery of depleted populations in the absence of fishing [3, 4]. Similar cases have also been observed for plant species. Lever et al. [5] suggested that pollinator populations may collapse suddenly once drivers of pollinator decline reach a critical point, and recovery subsequently requires quite strict conditions. In contrast, Allee effects are useful in other contexts, for example, in biological control of pests. An Allee effect can then be induced in the pest population by releasing sterile males [6], thereby promoting its suppression or obliteration.

Several mechanisms have been discussed that may give rise to Allee effects in animal populations. For example, limited reproductive output due to the difficulty of mating at low population densities in sexually reproductive species [7–9], lower survival possibilities due to reduced defense against predators [10, 11] and failure of catching prey in cooperatively hunting animals [12, 13]. Hence, in interactions of a population of consumers with its resource Allee effects can be expected to occur only if there is some density-dependent interaction among the consumer individuals and not when their competition for resources is purely exploitative.

In consumer-resource models, maturation, reproduction and mortality are the main drivers of consumer population growth. Recently, a number of studies have focused on the population dynamic consequences of resource-dependent development and maturation of juveniles in contrast to the more classic consumer-resource models that only account for resource-dependent reproduction (e.g., [14, 15]). Resource-dependent development affects the population dynamics in case of energetic asymmetry between juveniles and adults, which, for example, occurs when the two stages are feeding on different resources that have different productivities or when they feed on the same shared resource but with different efficiencies [14]. Furthermore, changing consumer mortality rate has complicated effects on the dynamics of a structured consumer-resource model because it will not only change the total biomass but also the fraction of individuals with different body sizes. One of the consequences is that biomass density of specific size classes may in fact increase when the mortality rate is increased [16]. Due to such biomass overcompensation in response to increased mortality that occurs in a stage-structured consumer-resource systems, an emergent Allee effect may occur in size-selective predators of the consumer [17]. But in the absence of predators Allee effects have up-to-now not been reported for stage-structured interactions between consumers and their resource for the simple reason that the models do not include sexual reproduction or cooperative behaviour among consumer individuals.

Whereas the stage-structured models discussed above assume continuous reproduction of adults, in many species dynamics and/or behaviour vary on a seasonal basis with seasonal reproduction being the most common case. Some species only reproduce during a very short time period each year, while their foraging on resources and their decline in abundance through mortality occurs continuously throughout the year. Recruitment to the population of a pulse of newly produced offspring represents a perturbation that may be sufficiently substantial to disrupt the population-dynamic effects of consumer stage structure. Nonetheless, Sun and de Roos [18] studied a stage-structured consumer-resource biomass model with seasonal reproduction of adults and showed that this semi-discrete model exhibits switches between a “development-controlled” state and a “reproduction-controlled” state similar to its analogue with continuous reproduction [16, 19]. However, it is not clear whether and how seasonal reproduction can influence the effects of increasing mortality rate of the consumer, such as biomass overcompensation.

In this paper we formulate a consumer-resource biomass model with seasonal reproduction of consumers and reveal how an Allee effect may arise as a consequence of density dependence in the timing of consumer maturation, which at low population density translates into a positive density-dependent influence on the reproductive output at the end of the season.

## Model formulation

Our model is identical to the model presented by Soudijn and de Roos [20], except for the fact that we assume the consumers to forage following a linear, type I instead of a type II functional response. Moreover, we analyse a scaled version of the model. The model accounts for one shared resource *R* and one consumer population. Following [20] we assume that the consumer individuals are distinguished by their body size, denoted by *s*, and all consumer individuals are born with the same body size *s*_b_ and can mature at any body size. The consumer population is thus divided into two stages: juvenile stage and adult stage.

Dynamics of the resource in the absence of consumers follow semi-chemostat growth with turn-over rate *ρ* and maximum density *K*_r_. The consumer population is represented with the biomass density of juvenile, *J*, and adult consumers, *A*, respectively. All consumer individuals forage on the resource and die due to background mortality and possibly starvation mortality. Juveniles are growing in body size, and adults do not grow any more but are accumulating their entire net-biomass production as reproductive energy storage *B*. These dynamics are considered to be continuous in time and taking place throughout the entire growing season. We hence refer to them as within-season dynamics. To reduce the number of parameters, we fix the duration of one season, which is also defined as the interval between two reproductive events, to 1. Furthermore, we denote *n* as the index of the growing season, and *t* ∈ (0,1) as the time within the *n*th season. In addition, we define *R*_*n*_(*t*), *J*_*n*_(*t*), *A*_*n*_(*t*) and *B*_*n*_(*t*) to be resource density, juvenile biomass, adult biomass and energy storage, respectively, at time *t* in the *n*th growing season. The dynamics of the model within the *n*th season is described by a set of ODEs that is shown in Table 1.

**Table 1:** Equations describing model dynamics within the *n*th growing season. Turn-over of resource, development of juveniles, allocation of energy storage for reproduction by adults and mortality of consumer individuals are assumed to take place continuously throughout the growing season. Individuals will experience starvation mortality rate when their net biomass productivity is not sufficient to cover their maintenance requirements, which occurs when *ν*_j_(*R*) < 0 or *ν*_a_(*R*) < 0. Maturation of juveniles and allocation to energy storage by adults cease when juveniles or adults starve, respectively.

Equations	Definition
$\frac{\text{d}R_{n}}{\text{d}t}=G\left(R_{n}\right)-I_{\max}R_{n}\left(J_{n}+\theta A_{n}\right)$	resource biomass dynamics
$\frac{\text{d}J_{n}}{\text{d}t}=\nu_{\text{j}}^{+}\left(R_{n}\right)J_{n}-\gamma \left(\nu_{\text{j}}\left(R_{n}\right),\mu \right)J_{n}-d_{\text{j}}\left(R_{n}\right)J_{n}$	juvenile biomass dynamics
$\frac{\text{d}A_{n}}{\text{d}t}=\gamma \left(\nu_{\text{j}}\left(R_{n}\right),\mu \right)J_{n}-d_{\text{a}}\left(R_{n}\right)A_{n}$	adult biomass dynamics
$\frac{\text{d}B_{n}}{\text{d}t}=\nu_{\text{a}}^{+}\left(R_{n}\right)A_{n}-d_{\text{a}}\left(R_{n}\right)B_{n}$	energy storage dynamics
*G*(*R*) = *ρ*(*K*_r_ − *R*)	turn-over of the resource
*ν*_j_(*R*) = *σI*_max_*R* − *Q*	net biomass production of juveniles
*ν*_a_(*R*) = *σθI*_max_*R* − *Q*	net biomass production of adults
$\nu_{\text{j}}^{+}\left(R\right)=\{\begin{array}{cc} \nu_{\text{j}}\left(R\right), & \text{if}\;\nu_{\text{j}}\geq 0\\ 0, & \text{otherwise} \end{array}$	somatic growth of juveniles
$\nu_{\text{a}}^{+}\left(R\right)=\{\begin{array}{cc} \nu_{\text{a}}\left(R\right), & \text{if}\;\nu_{\text{a}}\geq 0\\ 0, & \text{otherwise} \end{array}$	rate at which adults accumulate energy
$\gamma \left(\nu_{\text{j}}\left(R\right),\mu \right)=\{\begin{array}{cc} \frac{\nu_{\text{j}}\left(R\right)-\mu }{1-z^{1-\mu /\nu_{\text{j}}\left(R\right)}}, & \text{if}\;\nu_{\text{j}}>0\\ 0, & \text{otherwise} \end{array}$	maturation rate of juveniles*
$d_{\text{j}}\left(R\right)=\mu +\left(\nu_{\text{j}}^{+}\left(R\right)-\nu_{\text{j}}\left(R\right)\right)$	total per capita mortality rate of juveniles
$d_{\text{a}}\left(R\right)=\mu +\left(\nu_{\text{a}}^{+}\left(R\right)-\nu_{\text{a}}\left(R\right)\right)$	total per capita mortality rate of adults

* Mathematically, in the limit *ν*_j_ → *μ*, *γ*(*ν*_j_, *μ*) is approaching −*ν*_j_/ln(*z*). In our calculations, when *ν*_j_ is sufficiently close to *μ*, the maturation rate is set to −*ν*_j_/ln(*z*).

Juvenile and adult consumers forage on the resource following a linear functional response with attack rates per unit body mass equal *I*_max_ and *θI*_max_, respectively. Here *θ* is the adult-juvenile mass-specific intake ratio, reflecting the competitive ability in resource foraging of adults compared to juveniles. The ingested resource is converted to consumer biomass with an efficiency *σ*. Maintenance requirements per unit body mass of juveniles and adults are equal and denoted by *Q*. The net biomass productivity per unit body mass for juveniles and adults, denoted as *ν*_j_(*R*) and *ν*_a_(*R*), respectively, equals the balance between assimilation rate and maintenance requirements.

At low resource densities ingestion may be not sufficient to cover the maintenance requirements of consumer individuals, in which case they will start to experience starvation mortality. As discussed in [18], we assume that maturation of juveniles as well as the energy storage for reproduction by adults halts when juveniles and adults starve, respectively. We therefore introduce the notations $\nu_{\text{j}}^{+}\left(R\right)$ and $\nu_{\text{a}}^{+}\left(R\right)$ to restrict the net biomass productivities per unit body mass for juveniles and adults, respectively, to non-negative values. In case of starvation, juveniles and adults experience an increase in mortality rates equal $\left(\nu_{\text{j}}^{+}\left(R\right)-\nu_{\text{j}}\left(R\right)\right)$ and $\left(\nu_{\text{a}}^{+}\left(R\right)-\nu_{\text{a}}\left(R\right)\right)$, respectively. We assume the additional starvation mortality to exactly equal the (negative) net biomass production rate to ensure mass conservation in terms of juvenile and adult biomass, respectively, in case maintenance requirements are exceeding resource assimilation and consumers starve [21]. The total per-capita mortality rates of juveniles and adults thus equal the sum of the background and starvation mortality rate and are denoted by *d*_j_(*R*) and *d*_a_(*R*), respectively (see Table 1). Finally, the energy storage *B* experiences the same mortality rate as adults, that is *d*_a_(*R*), because energy storage accumulated by an adult perishes at the same time as the individual dies.

Throughout the growing season juvenile biomass increases through somatic growth of juveniles at mass-specific rate $\nu_{\text{j}}^{+}\left(R\right)$ and decreases through mortality at rate *d*_j_(*R*) and through maturation. Maturation of juveniles is modeled as in [18, 20] by a resource-dependent *per capita* maturation rate *γ*(*ν*_j_(*R*), *μ*) (see [20] for its derivation and justification). Here *μ* is the stage-independent background mortality rate of the consumer and *ν*_j_(*R*) is the mass-specific, net-biomass production rate. The exact form of the maturation rate is crucial for our current model to be a consistent approximation to the analogous fully size-structured version of the model, in which maturation of juveniles in each cohort occurs as a discrete event and new cohorts are formed at each reproduction event, because it ensures that the expected lifetime contribution of biomass per juvenile individual to the adult phase is the same in both models. Finally, this maturation rate ensures that the juveniles can only grow in body size and mature when they have positive net biomass productivity, that is when *ν*_j_(*R*) > 0, and takes into account that high mortality rate of juveniles may reduce the survival of juveniles till maturation [18], while also allowing juveniles to mature at any ages and body sizes [20].

In addition to the within-season dynamics shown in Table 1, consumer dynamics are also governed by adult reproduction, which occurs instantaneously at the beginning of each season. This pulsed reproduction event we refer to as the between-season dynamics. At the beginning of the (*n* + 1)th growing season adults release all the reproductive energy they have stored as offspring, thereby increasing juvenile biomass. As there is no change in resource density between seasons we just need to consider the change in consumer biomass due to the reproduction, which can be described by the following discrete map:

$$\begin{array}{c} R_{n+1}\left(0\right)=R_{n}\left(1\right), \end{array}$$ (1a)

$$\begin{array}{c} J_{n+1}\left(0\right)=J_{n}\left(1\right)+B_{n}\left(1\right), \end{array}$$ (1b)

$$\begin{array}{c} A_{n+1}\left(0\right)=A_{n}\left(1\right),\;B_{n+1}\left(0\right)=0, \end{array}$$ (1c)

where *R*_*n*_(1), *J*_*n*_(1), *A*_*n*_(1) and *B*_*n*_(1) represent the resource biomass, juvenile biomass, adult biomass and energy storage, respectively, at the end of the *n*th season. Note that Eq (1b) mathematically describes the releasing of the reproductive energy of adults, which comes from the consumption of resource, to juvenile stage. The full model, which classifies as a so-called semi-discrete population model, is described by the continuous dynamics defined in Table 1 in combination with the discrete-map defined by Eq (1).

## Model analysis

In this paper we are mainly focusing on the effect of varying consumer background mortality rate *μ* and the adult-juvenile intake ratio *θ* on the consumer biomass at equilibrium. While *μ* and *θ* will be varied all other parameters are given default values following [18]. The definitions and default values of all parameters are given in Table 2.

**Table 2:** Parameters and their default values.

Parameter	Unit	Value	Definition
*ρ*	per unit time	10	turn-over rate of the resource
*K*_r_	gram/L	2	maximum resource density
*θ*	-	varied	adult-juvenile mass-specific intake ratio
*I*_max_	per unit time	100	resource attack rate of consumer per unit body mass
*Q*	per unit time	10	maintenance cost of consumer per unit body mass
*σ*	-	0.5	conversion efficiency of consumer
*μ*	per unit time	varied	stage-independent background mortality rate of consumer
*z*	-	0.1	body size ratio of newborn and adult consumer

We numerically studied the model dynamics using C-based software for the integration of ordinary differential equations and calculated the stable and unstable equilibria of the model using standard root-finding procedures also implemented in C [22] to locate the fixed points of the equations: *R*_*n*+1_(0) = *R*_*n*_(1) = *R*_*n*_(0), *J*_*n*+1_(0) = *J*_*n*_(1)+*B*_*n*_(1) = *J*_*n*_(0) and *A*_*n*+1_(0) = *A*_*n*_(1) = *A*_*n*_(0), where *R*_*n*_(1), *J*_*n*_(1), *A*_*n*_(1) and *B*_*n*_(1) are calculated from *R*_*n*_(0), *J*_*n*_(0), *A*_*n*_(0) and *B*_*n*_(0) = 0 by numerical integration of the within-season dynamics. As a key feature of our model, however, it should be noted that due to the pulsed reproduction resource density, juvenile and adult biomass are always varying during a growing season as opposed to the constant equilibrium densities in continuous-time models. Additionally, we will not only show the equilibrium values that only reflect the biomass densities just after reproduction pulses at the beginning of each growing season, but will also show the composition of consumer biomasses throughout the entire season. Because of these, we focus on the average biomass of juvenile and adult consumers at equilibrium. These average densities were calculated by numerical integration of the ODEs $\text{d}\bar{J}/\text{d}t=J\left(t\right)$, $\text{d}\bar{A}/\text{d}t=A\left(t\right)$ and $\text{d}\bar{B}/\text{d}t=B\left(t\right)$ within each growing season with initial states $\bar{J}=\bar{A}=\bar{B}=0$. The programs used for calculations and generating the figures can be found from S1 File.

## Results

We study model dynamics with default values of parameters and use time series of these dynamics to show how the energetic asymmetry, caused by the varying adult-juvenile intake ratio *θ*, affects the consumer biomass. In addition, we analyse the average consumer biomass at equilibrium as a function of the varying stage-independent mortality rate *μ* and the adult-juvenile intake ratio *θ*. The increasing mortality rate *μ* can relax the competition among consumer individuals, leading to biomass overcompensation in one specific stage and an Allee effect when consumer biomass is close to the level of extinction. These results are shown by both one-parameter and two-parameter analysis.

### Different adult-juvenile intake ratio determines the energetic asymmetry

**Fig 1:**
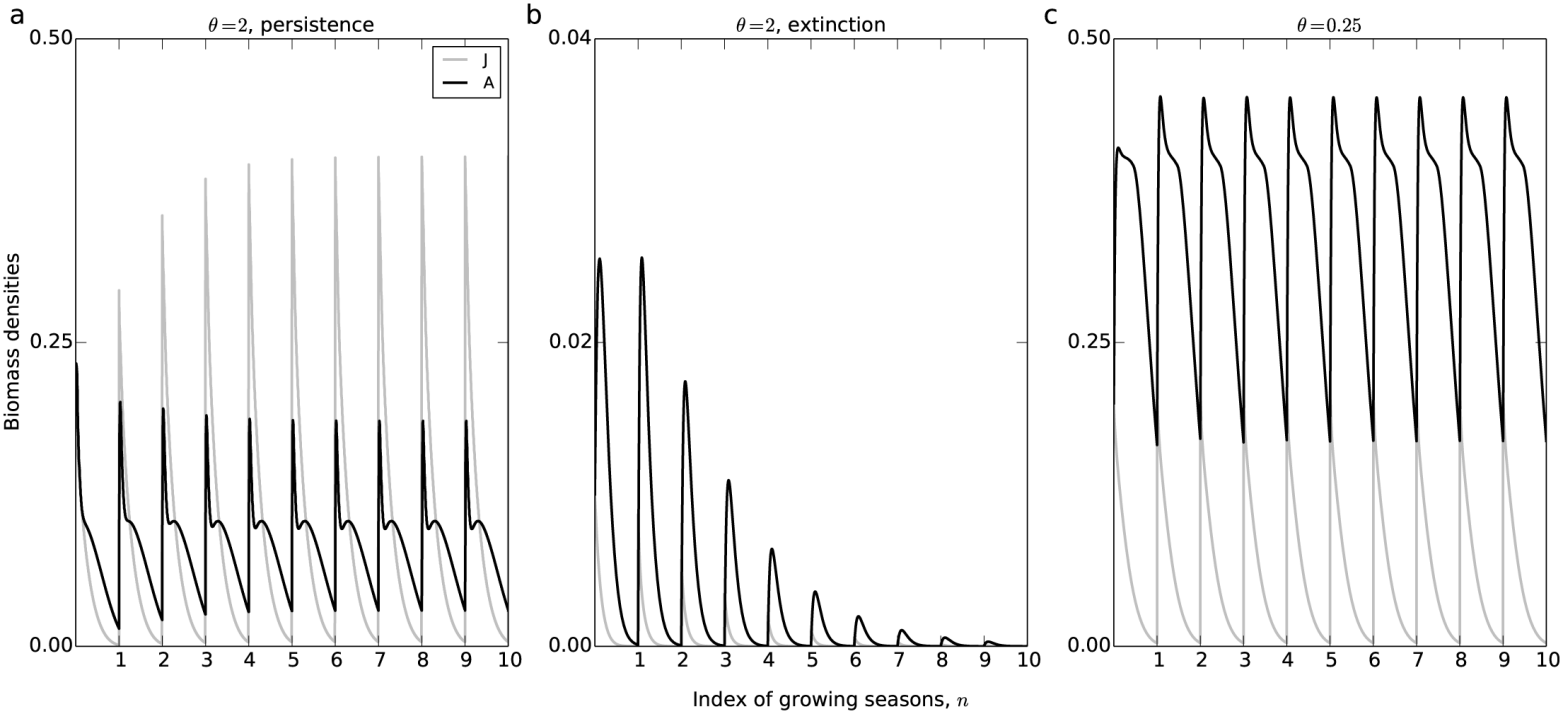
Time series of model dynamics. Time series of model dynamics for *θ* = 2 and *μ* = 9 (panels a and b) and *θ* = 0.25 and *μ* = 3.5 (panel c), showing juvenile (grey curves) and adult biomass (dark curves). In all panels, *R*_1_(0) = 1 and *B*_1_(0) = 0. In panels a and c, *J*_1_(0) = *A*_1_(0) = 0.2, while in panel b, *J*_1_(0) = *A*_1_(0) = 0.01. In all the panels, other parameters have their default value shown in Table 2.

As introduced before, *θ* reflects the competitive ability of adults compared to juveniles while foraging on the resource. When *θ* > 1 adults have a higher intake rate than juveniles, which implies that juveniles are more limited by the resource, and vice versa. Fig 1 illustrates the model dynamics for two different values of *θ*. In Fig 1a, *θ* = 2 and the initial biomass densities of juveniles and adults are high, which results in consumer persistence. Furthermore, the intake rate of juveniles is much lower than adults, resulting in a very low maturation rate and very few juveniles recruiting to the adult stage. Consequently, juvenile biomass is higher than adult biomass. Such a condition is referred to as a development-controlled population state, in which the model is governed by limited maturation of juveniles [18, 23]. In Fig 1b, *θ* is also set to 2 but the initial densities of juveniles and adults are low, which results in consumer extinction. For *θ* = 2 the model thus exhibits an Allee effect. In contrast, for *θ* = 0.25 (Fig 1c) an Allee effect does not occur. For these parameters juveniles have a much higher intake rate than adults and therefore mature fast into the adult stage. However, adults only store very little energy and reproduce few offspring. Consequently, adult biomass is much higher than juvenile biomass. This condition is referred to as a reproduction-controlled population state, in which the model is governed by limited reproduction of adults.

Fig 1 also shows that adult biomass increases abruptly after the reproduction. Just after reproduction juvenile biomass and resource density are both high, and therefore many individuals recruit to the adult stage. The time delay between the maturation of juveniles and the reproduction of adults is in fact too tiny to be observed in Fig 1. Finally, for both values of *θ* model dynamics converge to stable fixed-points (bistability when *θ* = 2 and a unique stable state when *θ* = 0.25), where all biomass densities at the same time points within each season are the same. In the following we study the changes in this stable fixed-point dynamics (which we will also refer to as an equilibrium) with parameters.

### Increasing background mortality rate results in an Allee effect

**Fig 2:**
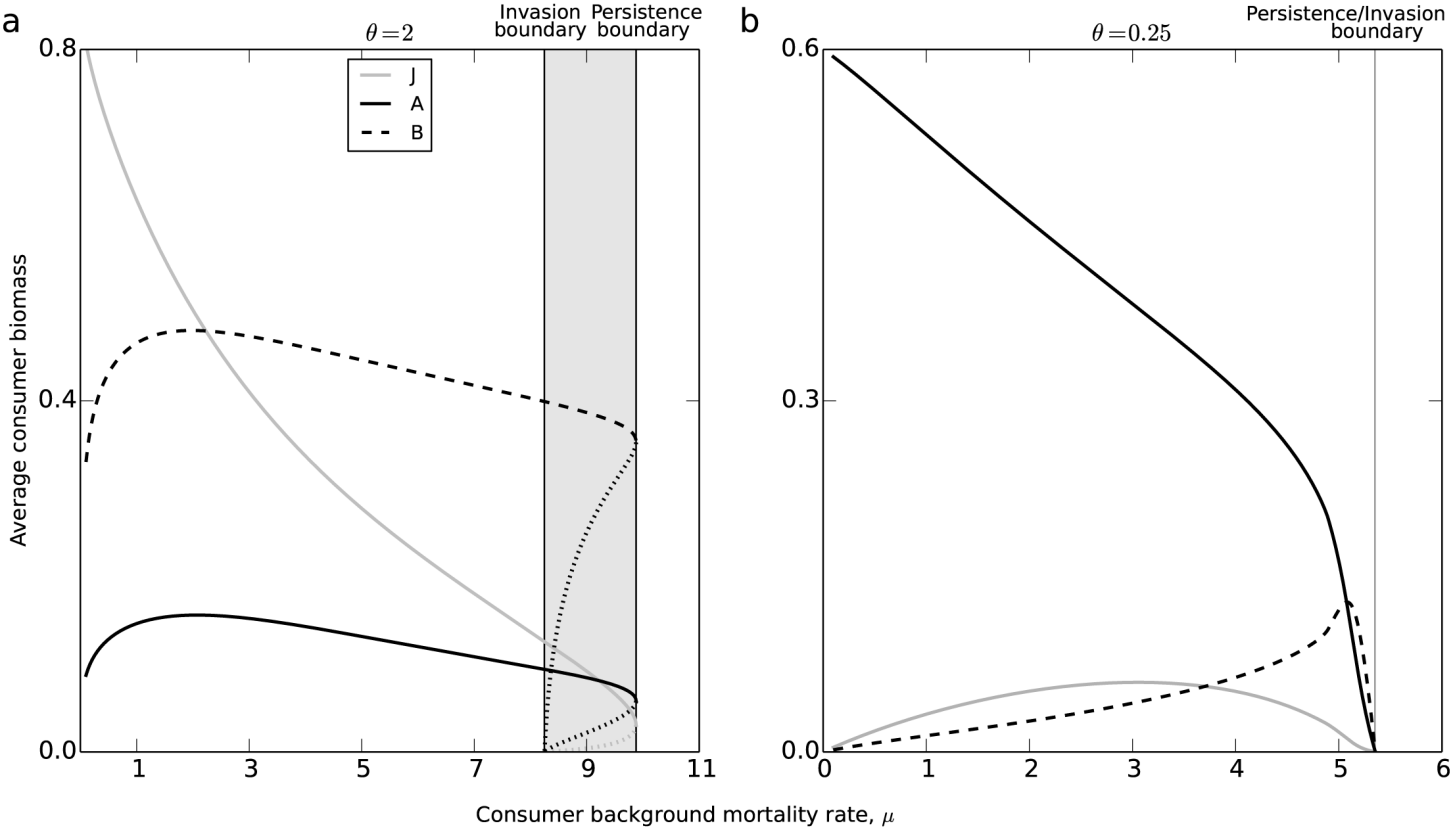
Bifurcation diagram. Average consumer biomass over the interval between 2 reproduction pulses as a function of the consumer background mortality rate *μ* when *θ* = 2 (panel a) and *θ* = 0.25 (panel b). Average juvenile biomass $\bar{J}$ (grey solid curves), average adult biomass $\bar{A}$ (dark solid curves) and average energy storage $\bar{B}$ (dark dashed curves) are shown. The persistence boundaries indicate the critical values of *μ* above which the consumer population cannot persist, the invasion boundaries indicate the values of *μ* above which the consumer cannot invade a resource-only equilibrium. The marked grey region, between these two boundaries in panel a indicates the region for which bistability occurs between a stable consumer-resource and a stable resource-only equilibrium, and the dotted curves in the bistability region indicate unstable equilibrium states. No bistability is observed in panel b. Other parameters have their default value shown in Table 2.

Fig 2 shows a bifurcation diagram of the average consumer biomass at equilibrium as a function of the consumer background mortality rate *μ*. The effects of increasing mortality rate *μ* are different for the two values of *θ*. For *θ* = 2 juveniles are more limited by the resource and they hence make up the majority of consumer biomass (Fig 2a). An increase in *μ* from its default value of *μ* = 0.1 decreases juvenile biomass, thereby relaxing the maturation bottleneck in the juvenile stage. The latter leads to a slight increase in adult biomass, as well as in energy storage, a phenomenon known as adult biomass overcompensation [23]. With further increases of *μ*, however, the negative effect of increasing mortality rate outweighs the positive effect of relaxing the juvenile maturation bottleneck. As a consequence, when μ⪆2, increasing *μ* only results in decreases in juvenile and adult biomasses and energy storage. A minor increase of the mortality rate above the persistence boundary (defined as the highest mortality level at which consumers manage to persist), which occurs around *μ* ≈ 10, results in a collapse of the consumer population from a considerable biomass density to 0. For mortality rates close to the persistence boundary, the energy storage makes up the majority of the consumer biomass. But since the storage is inert from the point of view of producing new biomass, a tiny increase in background mortality rate has the potential to drive the consumer population to extinction. The marked grey parameter region at high mortality rates represents the parameter range, for which the consumer population will go extinct, when reaching low biomass densities and can not increase from these low densities in the resource-only environment. Bistability hence occurs between equilibria with and without consumers as a consequence of reduced consumer growth at low densities, representing an Allee effect.

The effect of increasing background mortality is different when juveniles have a higher intake rate than adults. Fig 2b shows bifurcation diagrams for *θ* = 0.25, for which value adults are more limited by the resource and make up the majority of the consumer biomass. As *μ* increases the bottleneck in adult stage is relaxed leading to increasing energy storage and overcompensation in juvenile biomass. Adult biomass is decreasing when *μ* increases. The consumer population goes extinct at *μ* ≈ 5.3, the persistence boundary. In contrast to the case for *θ* = 2 shown in Fig 2a no bistability is observed for *θ* = 0.25. The reason is that for such a small *θ* adults cannot store much reproductive energy even if the resource availability is abundant. Therefore, in Fig 2b, energy storage only makes up a small part of the consumer biomass. Hence, the perturbation in consumer biomass due to the seasonal reproduction is tiny and the model is quite close to a continuous-time one, in which the Allee effect has been shown not to occur [16].

### Larger adult-juvenile mass-specific intake ratio increases the likelihood of consumer collapse

**Fig 3:**
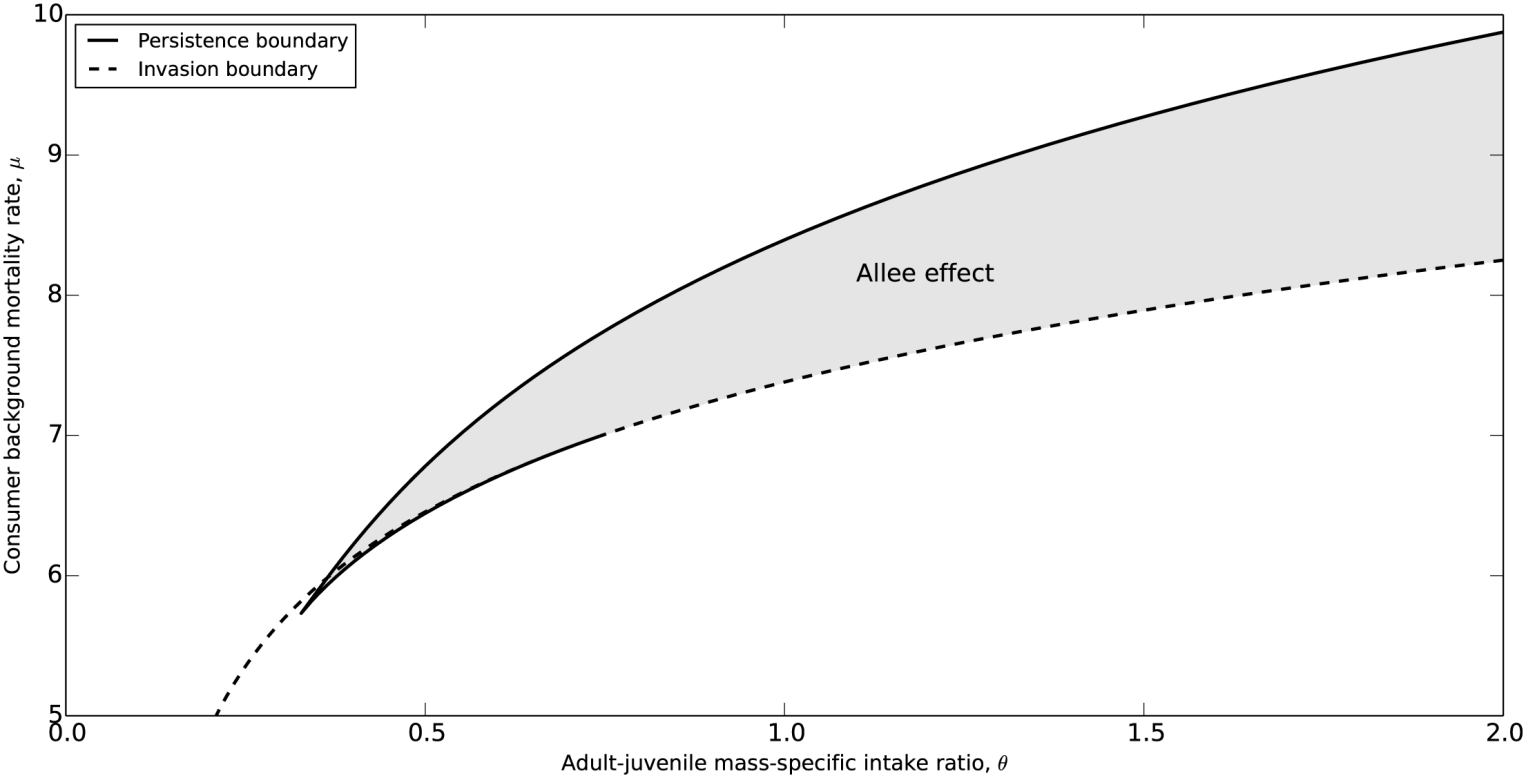
Two-parameter bifurcation diagram. Combinations of the adult-juvenile intake ratio *θ* and the consumer background mortality rate *μ* for which the consumer can persist and for which bistability occurs. Solid and dashed curves represent the persistence and invasion boundaries as a function of the two parameters, respectively. Bistability occurs in the grey parameter region, corresponding to the marked regions shown in Fig 2, while consumer persistence is furthermore possible in the parameter region below the dashed curve. All other parameters have their default value shown in Table 2.

Fig 3 shows how the occurrence of the Allee effect depends on different values of adult-juvenile intake ratio *θ* and consumer background mortality rate *μ*. For θ⪅0.33 bistability does not occur in the model, the invasion boundary is thus identical to the persistence boundary; For 0.33⪅θ⪅0.75 there is a very small parameter region in which two different consumer-resource equilibria occur as alternative stable states (ASS), but this domain is so tiny that it can for all practical purposes be ignored. Bistability between a consumer-resource equilibrium state and a resource-only equilibrium state, in which consumers fail to invade at low population densities, and the associated Allee effect therefore mainly occurs in a considerable parameter range with larger values of the adult-juvenile intake ratio *θ*.

At higher *θ* values both the persistence and the invasion boundary appear at higher values of background mortality *μ*, but the distance between them increases as well. The parameter region with bistability and an Allee effect hence expands with increases in *θ*. For larger *θ* values adults have a higher mass-specific intake rate and are hence less resource-limited than juveniles. In this case, adult individuals can store more reproductive energy, resulting in a more significant perturbation in consumer biomass due to the seasonal reproduction. At higher *θ* values the seasonality in consumer dynamics is hence stronger, which is a necessary condition for the Allee effect to occur. Therefore, with larger differences in mass-specific intake rate between adults and juveniles the likelihood that a consumer population will fail to recover and go extinct after a population decline due to overexploitation will increase, even though in principle the consumer population can face higher mortality levels while still persisting.

### Mechanisms at the individual level: The timing of maturation

Since individuals stop growing at maturation and afterward store their net-energy production for reproduction, the timing of maturation is crucial for the growth of juvenile individuals and their subsequent fecundity as adult. To elucidate the individual-level mechanisms generating the Allee effect, we focus on the life history of one newborn consumer for *θ* = 1 and *μ* = 8, for which an Allee effect occurs (Fig 3). The occurrence of the Allee effect implies that the consumer population persists when resource densities are suppressed by consumer foraging, while it goes extinct when the resource is at its maximum density, unaffected by consumer grazing. We compare the fate of a single consumer in these two cases: when the consumer population through foraging exerts a feedback on the resource density (coupled case) and when the resource is at its maximum density (decoupled case). In the coupled case we integrate model dynamics until it reaches stable fixed-point dynamics, while in the decoupled case we merely assume that *R* = *K*_r_ and constant. Following this we introduce one unit of juvenile biomass into the model, assuming that the introduced individual does not affect the resource availability. We focus on the resource availability and the consumer biomass during the season, and the expected reproductive output at the end of the season, denoted by *R*_0_, for consumer individuals that mature at time *t*_m_ within the growing season.

**Fig 4:**
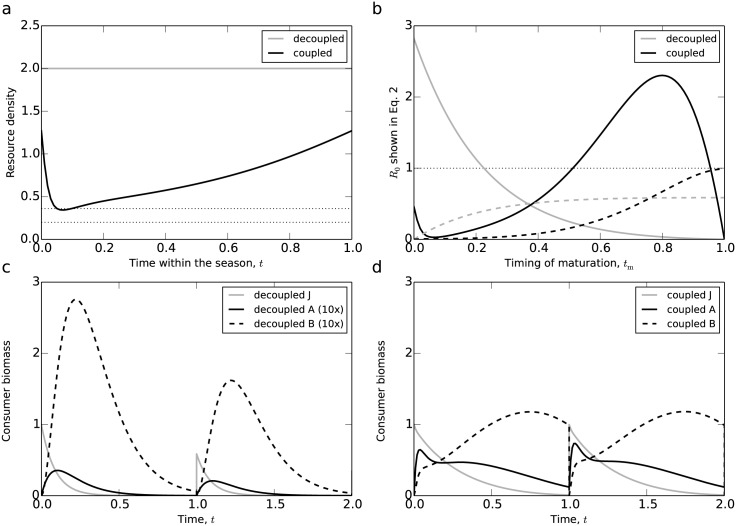
Mechanisms. Panel a: resource densities in the presence (coupled case, dark curve) and absence (decoupled case, grey curve) of foraging feedback of consumers on the resource, which translates into consumer density dependence. The two horizontal dotted curve indicates the critical values of resource availability below which the juvenile consumers will starve (bottom, that is *R* = *Q*/(*σI*_max_)) and below which maintenance and background mortality losses exceed the juvenile net biomass productivity (top, that is *R* = (*Q* + *μ*)/(*σI*_max_)), respectively; b: the contribution to the reproductive output at the end of the season by the individuals that mature at time *t*_m_ in the growing season, *R*_0_ (see Eq (2)), as a function of the timing of maturation *t*_m_ in the coupled case (dark solid curve) and the decoupled case (grey solid curve). The dark dashed curve and the grey dashed curve show the cumulative value of *R*_0_ (calculated as $\int_{0}^{t_{\text{m}}}\;R_{0}\left(\tau \right)\text{d}\tau$ with initial value *R*_0_(0) = 0) in the coupled and decoupled case, respectively. The horizontal dotted curve indicates the threshold of *R*_0_ = 1; c: the consumer biomass during the season in the decoupled case. Note that in this panel the densities of adult biomass and reproductive storage have been scaled down by a factor of 10 for graphical purposes; d: the consumer biomass during the season in the coupled case. Here *θ* = 1 and *μ* = 8, and all other parameters have their default value shown in Table 2.

The resource densities in both decoupled and coupled case are shown in Fig 4a. In the coupled case, the resource availability is decreasing in a short period at the beginning of the season and after that recovers until the end of the season. Because of the stable fixed-point dynamics considered the resource availabilities at *t* = 0 and *t* = 1 are equal. In the coupled case the resource availability is always much lower than in the decoupled case, where *R* is continuously at its maximum level *K*_r_. Furthermore, in both cases the resource availabilities are higher than the starvation threshold so the juveniles and adults never starve within the season (as shown by the bottom horizontal dotted curve).

The contribution to the reproductive output at the end of the season by the individuals that mature at time *t*_m_ in the growing season is given by

$$\begin{array}{c} R_{0}\left(t_{\text{m}}\right)=\gamma \left(\nu \left(R\left(t_{\text{m}}\right)\right),\mu \right)\text{exp}\left(\;-\mu +\;\int_{0}^{t_{\text{m}}}\;\;\;\nu \left(R\left(\tau \right)\right)-\gamma \left(\nu \left(R\left(\tau \right)\right),\mu \right)\text{d}\tau \right)\int_{t_{\text{m}}}^{1}\;\;\;\nu \left(R\left(\tau \right)\right)\text{d}\tau . \end{array}$$ (2)

The derivation of this expression for *R*_0_ can be found from S1 Appendix. We computed the value of *R*_0_ as a function of *t*_m_ by differentiating the above expression with respect to *t*_m_ and numerically integrating the resulting ordinary differential equation together with the differential equation system describing the within-season consumer-resource dynamics (cf. Table 1; see [24] for an explanation of this approach). Notice that the function *R*_0_(*t*_m_) takes into account the probability that a consumer individual matures at time *t*_m_, its size at maturation, its subsequent capacity to accumulate reproductive energy and its probability to survive till the end of the growing season.

The expected reproductive output *R*_0_ as a function of *t*_m_ is shown in Fig 4b. For both the coupled and decoupled case *R*_0_ equals 0 if *t*_m_ = 1, because individuals that mature at the end of the season have no time to accumulate reproductive energy at all. In the decoupled case *R*_0_ is a decreasing function of *t*_m_, dropping below 1 for $t_{\text{m}}⪆0.24$, mainly because individuals in this case mature early in the season as a consequence of the high resource density. In the coupled case *R*_0_ decreases from *t*_m_ = 0 until *t*_m_ ≈ 0.08, after which it recovers and exceeds the value of 1 in the range $0.5⪅t_{\text{m}}⪅0.95$. The value of *R*_0_ reaches its maximum level for *t*_m_ ≈ 0.8. Hence, in the coupled case individuals tend to mature later at larger body sizes. These results show that there exists a trade-off between maturation at a larger body size (mature later) and accumulating reproductive energy for a longer period (mature earlier). The overall expected reproductive output of a newborn consumer individual at the first reproduction event after its birth equals the integral of *R*_0_ from *t*_m_ = 0 to *t*_m_ = 1 (as shown by the dashed curves in Fig 4b), as this properly weighs the expected output of an individual that matures at *t*_m_ and its probability to mature at this time within the season. As shown by the two dashed curves, in the coupled case the cumulative value of *R*_0_ from *t*_m_ = 0 to *t*_m_ = 1 is much higher than that in the decoupled case (because at *t*_m_ = 1 the dark dashed curve has a much higher value than the grey dashed one). Fig 4b makes clear that despite the consistently lower resource density the expected reproductive output is higher in the coupled case than in the decoupled case, because of the delayed maturation at larger body sizes. It is this difference in the timing of maturation that makes that the consumer can persist at the high mortality level *μ* = 8 when at high biomass density, depressing the resource density, whereas it fails to grow at low biomass density when resource density is high.

To elucidate the mechanism generating the Allee effect in even more detail, we compare the dynamics of the one unit of juvenile biomass in the decoupled and coupled case (Fig 4c and 4d). At high resource density in the decoupled case all juveniles mature into adult stage before *t* ≈ 0.4, while in the coupled case the maturation is delayed due to density dependent feedback of the population on the resource. The energy storage, in the decoupled increases rapidly to a high peak at *t* ≈ 0.2 within each season and then it decreases due to low adult biomass and mortality (Fig 4c), while in the coupled case it increases until a much lower peak at *t* ≈ 0.75 within each season and then decreases slowly to a much higher value at the end of the season (Fig 4d). As a consequence, after the reproduction at the beginning of the next season, in the coupled case juvenile biomass can always recover to the initial level, whereas in the decoupled case the juvenile biomass becomes lower and lower, indicating that the consumer population can only persist in the coupled case. Furthermore, the net biomass productivity of consumers outweighs the mortality during the largest part of the cycle for both juveniles and adults. However, the net-production of juveniles translates into larger body masses that further increase the use of resources and hence production of new biomass, whereas the net-production of adults is allocated to storage that is inert from a point of view new biomass production. This results in the effect that delayed maturation (coupled case) in favour of somatic growth is more favourable under conditions of high mortality compared to early maturation at smaller body sizes (decoupled case), also because larger adults that have matured later reach a higher per-capita fecundity, since the latter is proportional to body size.

## Discussion

In analogous consumer-resource models, in which individuals mature at reaching a threshold body size and reproduce continuously following maturation, an Allee effect can not occur with an increase in the background mortality rate [16, 19]. In these continuous-time models consumers are assumed to interact with each other only through purely exploitative competition for food. Consumers hence exhibit none of the previously identified mechanisms that give rise to an Allee effect, such as group foraging or group defence against predators. Although the current semi-discrete model does not incorporate any of these between-consumers interactions either an Allee effect occurs. We have shown that the Allee effect only occurs in our semi-discrete biomass model for large values of adult-juvenile intake ratio (*θ*) when consumer dynamics are strongly seasonal, and the likelihood of the occurrence of the Allee effect and population collapses increases with *θ* (Fig 3). Furthermore, we have shown that the Allee effect occurs as a result of the pulsed reproduction in combination with the resource-dependent maturation rate, rather than the mechanisms reported before. The resource-dependent growth rate and rate of energy accumulation for reproduction give rise to the classical life-history trade-off between growth and reproduction [25, 26]. These findings contrast with stage-structured consumer-resource models with continuous reproduction and results as a consequence of the timing of maturation of the juveniles born in a particular reproductive event.

The timing of maturation is important because it is associated with a trade-off between early and delayed maturation, ensuring either high survival until maturation or a larger body size and higher subsequent fecundity [27, 28]. Classic theories predict that early maturation at smaller sizes is advantageous in an environment with high mortality risk because the species can benefit from a higher survival possibility before reproduction, rather than a larger size at maturation [25, 29]. There has also been evidence showing that species that mature later at larger sizes are more vulnerable at given mortality rates compared to their nearest relatives that mature earlier [30]. However, our results show that the consumer population will benefit from later maturation. In our model, the resource-dependent maturation rate makes that individuals mature early when resource density is high, whereas at lower resource densities that are suppressed by consumer feeding individuals mature later but at larger body sizes, by which they ultimately realise a larger reproductive output. The results are quite different from the ones shown in [31] and [32], in which the authors suggested that the individuals that mature later have lower reproductive success and early maturation does not compromise the reproductive output, respectively.

The maturation strategies of species have important implications in many fields of ecology in particular in fishery managements. Extensive empirical studies have shown that size-selective fishing mainly targeting on large, adult individuals has resulted in smaller sizes at maturation in marine fishes [33, 34]. Such changes result in significant declines of fish stock and even collapses of fish populations [35], as well as huge economic losses [36]. During the past two decades, the fishery industry has adopted several strategies to reduce the loss, one of which is to select breeders to produce later-maturation offspring [37]. However, the effectiveness of these management strategies is still unclear [38], and the recovery of fish stock at low abundances seems to be quite difficult because smaller sizes at maturation have the potential to result in failure of recovery [39]. Our current study provides a theoretical insight into these empirical results by showing the advantages of later maturation at larger body sizes, even if experienced mortality is substantial and population densities are low.

Although our results pertain to a model with a single consumer and resource population, they are likely to generalize to more realistic systems. For example, the Allee effect results from the density-dependence in juvenile maturation, which delays their maturation till just before the next reproduction event. This density-dependent delay may occur even when juveniles and adults feed on entirely different resources, as long as the impact of juvenile foraging on their exclusive resource is sufficiently high.

The direct consequence of an Allee effect and sudden collapses in stage-structured consumer populations with seasonal reproduction is that these dynamic phenomena may impact the structure of larger communities, because they may affect the persistence of predators at higher level when predation is intense. When the consumer population is suppressed to very low densities by predation the risk of a sudden collapse will increase [40, 41], which may ultimately result in a decrease in the density of the predators. Such effects may also be exacerbated by human activities, i.e., by over-exploitation. Therefore, it is of great importance to examine the threshold of population density under which Allee effects can occur, avoiding unexpected extinction of species and the loss of biodiversity.

In contrast, the Allee effect can also affect the possibility of establishment of an invading species. Generally, the success of invasion depends on a positive relationship between probability of establishment and the numbers of individuals introduced [42, 43]. Due to the Allee effect this relationship becomes non-linear, and it is suggested that the pest population will be eradicated when its density is suppressed to a level below the Allee effect threshold [44]. Seasonal reproduction may thus reduce the possibility of invasion because it can generate Allee effects in the structured invaders. In other words, the invasion can be prevented by creating Allee effects in seasonally reproducing invaders to protect the local species and ecosystems.

## Supporting information

S1 Appendix Derivation of *R*_0_ in Eq (2). (PDF)

S1 File Computational programs. The programs used for fixed-point analysis and the MATLAB script for generating the bifurcation diagrams (Figs 2 and 3).
(ZIP)
