# Supplementary material for: Seasonal reproduction leads to population collapse and an Allee effect in a stage-structured consumer-resource biomass model when mortality rate increases
Source: PLoS One. 2017 Oct 31;12(10):e0187338. doi: 10.1371/journal.pone.0187338 (PMC5663510; doi:10.1371/journal.pone.0187338)
Supplement: S1 Appendix — (PDF) [file pone.0187338.s001.pdf]

Supplementary material to Sun and De Roos: Seasonal reproduction leads to population collapse and an Allee effect in a stage-structured consumer-resource biomass model when mortality rate increases

Here we present the derivation of R_0 shown in Eq. 2 in the main text.

For $\theta = 1$ juveniles and adults have the same net biomass productivity. To simplify the discussion we denote the net biomass productivity of the consumer individuals as $\nu(R)$, defined as:

$$\nu(R) = \sigma I_{\max} R - Q, \tag{1}$$

Because consumers never starve during the season (Figure 4a), the mortality rates of juveniles and adults are also the same and equal to μ. The probability, denoted by $P_{\mathrm{j}}(t)$, that the consumer individual is still in the juvenile stage at time t follows a dynamics described by the following ordinary differential equation:

$$\frac{\mathrm{d}P_{\mathrm{j}}}{\mathrm{d}t} = \left(-\gamma\left(\nu\left(R\left(t\right)\right), \mu\right) - \mu\right) P_{\mathrm{j}}, \quad P_{\mathrm{j}}(0) = 1. \tag{2}$$

This ODE can be formally solved to yield as solution:

$$\begin{aligned} P_{\mathrm{j}}(t_{\mathrm{m}}) &= \exp\left(\int_0^{t_{\mathrm{m}}} -\mu - \gamma\left(\nu\left(R\left(\tau\right)\right), \mu\right) \mathrm{d}\tau\right) \\ &= \exp\left(-\mu t_{\mathrm{m}}\right) \exp\left(-\int_0^{t_{\mathrm{m}}} \gamma\left(\nu\left(R\left(\tau\right)\right), \mu\right) \mathrm{d}\tau\right), \end{aligned} \tag{3}$$

The maturation rate at time t_{m} out of the juvenile stage is then given by

$$h(t_{\mathrm{m}}) = \gamma\left(\nu\left(R\left(t_{\mathrm{m}}\right)\right), \mu\right) P_{\mathrm{j}}(t_{\mathrm{m}}). \tag{4}$$

If we would consider a cohort of N newly produced offspring, $h(t_{\mathrm{m}}) \cdot N$ would represent the expected rate of maturation of these individuals at time t_{m}.

The underlying assumption in the model about consumer life history [1, 2] is that juvenile individuals are born at the beginning of a season with body size $s(0) = s_{\mathrm{b}}$, which we set without loss of generality equal to 1. They subsequently grow following:

$$\frac{\mathrm{d}s}{\mathrm{d}\tau} = \nu\left(R\left(\tau\right)\right) s, \quad s(0) = s_{\mathrm{b}}.$$

Individual body size at t_{m} hence equals:

$$s(t_{\mathrm{m}}) = \exp\left(\int_0^{t_{\mathrm{m}}} \nu\left(R\left(\tau\right)\right) \mathrm{d}\tau\right). \tag{5}$$

In the period from t_{m} to the end of the season an individual maturing at t_{m} accumulates and stores reproductive energy at a rate $s(t_{\mathrm{m}})\, \nu(R(\tau))$, which is proportional to its size at maturation. The contribution to the reproductive output at the end of the season by the individuals that mature at time t_{m} in the growing season is thus given by

$$
\begin{aligned}
R_0\left(t_{\mathrm{m}}\right) &= h\left(t_{\mathrm{m}}\right) \int_{t_{\mathrm{m}}}^{1} s\left(t_{\mathrm{m}}\right) \nu\left(R\left(\tau\right)\right) \mathrm{d}\tau \cdot \exp\left(-\mu\left(1-t_{\mathrm{m}}\right)\right) \\
&= h\left(t_{\mathrm{m}}\right) s\left(t_{\mathrm{m}}\right) \int_{t_{\mathrm{m}}}^{1} \nu\left(R\left(\tau\right)\right) \mathrm{d}\tau \cdot \exp\left(-\mu\left(1-t_{\mathrm{m}}\right)\right).
\end{aligned} \tag{6}
$$

Using Equations (3)-(5) this expression can be rewritten as:

$$
R_0\left(t_{\mathrm{m}}\right) = \gamma\left(\nu\left(R\left(t_{\mathrm{m}}\right)\right), \mu\right) \exp\left(-\mu + \int_0^{t_{\mathrm{m}}} \nu\left(R\left(\tau\right)\right) - \gamma\left(\nu\left(R\left(\tau\right)\right), \mu\right) \mathrm{d}\tau\right) \int_{t_{\mathrm{m}}}^{1} \nu\left(R\left(\tau\right)\right) \mathrm{d}\tau. \tag{7}
$$

References

[1] De Roos AM et al. 2008 Simplifying a physiologically structured population model to a stage-structured biomass model. *Theor. Popul. Bio.* **73,** 47–62. (doi:10.1016/j.tpb.2007.09.004)

[2] Soudijn FH, de Roos AM. 2017 Approximation of a physiologically-structured population model with seasonal reproduction by a stage-structured biomass model. *Theor. Ecol.* **10,** 73–90. (doi:10.1007/s12080-016-0309-9)
