# Supplementary material for: Seasonal reproduction leads to population collapse and an Allee effect in a stage-structured consumer-resource biomass model when mortality rate increases
Source: PLoS One. 2017 Oct 31;12(10):e0187338. doi: 10.1371/journal.pone.0187338 (PMC5663510; doi:10.1371/journal.pone.0187338)
Supplement: S1 File — The programs used for fixed-point analysis and the MATLAB script for generating the bifurcation diagrams (Figs 2 and 3). (ZIP) [file pone.0187338.s002.zip › Fig2and3/README.docx]

**Program for calculations**

The programs for calculations and generating the Figure 2 and 3. We use standard root-finding procedures implemented in C to study the equilibrium as a function of one and two parameters.

To study the fixed-point dynamics, with the Makefile the model can be complied on the command-line on Mac OS X Yosemite. The detail of this method can be found from <https://bitbucket.org/amderoos/findcurve>. The file Cja.c contains three calculation types: one parameter fixed-point dynamics (Cja), two-parameter limit point continuation (Cja_lp) and two-parameter branch point continuation (Cja_bp). The latter two can be created by establishing soft links to the Cja.c file. The code for calculating are provided at the beginning of the file Cja.c and can be directly implemented on the command-line. Cja_lp.c and Cja_bp.c are respectively used for finding the persistence boundary and the invasion boundary. The .cvf files give the parameter settings for the calculation: eq20 and eq025 mean the left and the right panel of Figure 2; lp and bp are corresponding to Cja_lp.c and Cja_bp.c. The results will be stored in the .out files and can be plotted with the script Fig2and3.m using Matlab.
